# Risque transfusionnel en Afrique subsaharienne: état des lieux et défis spécifiques à la République démocratique du Congo

**DOI:** 10.48327/mtsi.v5i4.2025.693

**Published:** 2025-09-25

**Authors:** Lambert Morisho MULAKWA, Archippe Muhandule BIRINDWA, Chérone Nancy Mbani MPEGA NTIGUI, Patrick Ntagereka BISIMWA, Sandrine Lydie OYEGUE LIABAGUI

**Affiliations:** 1 École doctorale régionale d’Afrique centrale en infectiologie tropicale de Franceville, Université des sciences et techniques de Masuku (USTM), BP: 876 Franceville, Gabon; 2 Laboratoire de biologie moléculaire, Faculté de médecine et santé communautaire, Université évangélique en Afrique (UEA), BP: 3323 Bukavu, RDC; 3 Faculté de médecine et des sciences de la santé, Université des sciences et techniques de Masuku (USTM), BP: 914 Franceville, Gabon; 4 Département de biologie, Faculté des sciences, Université des sciences et techniques de Masuku (USTM), BP: 914 Franceville, Gabon; 5 Unité Évolution, épidémiologie et résistances parasitaires, Centre interdisciplinaire de recherches médicales de Franceville (CIRMF), BP: 769 Franceville, Gabon

**Keywords:** Sécurité transfusionnelle, Infections post-transfusionnelles, Hémovigilance, Systèmes de santé, République démocratique du Congo, Afrique subsaharienne, Transfusion Safety, Post-Transfusion Infections, Hemovigilance, Health Systems, Democratic Republic of Congo, Sub-Saharan Africa

## Abstract

**Introduction:**

La sécurité transfusionnelle demeure un défi sanitaire majeur en Afrique subsaharienne (ASS), particulièrement en République démocratique du Congo (RDC), où l’instabilité politique, la faiblesse des infrastructures et l’absence de systèmes d’hémovigilance intégrés augmentent les risques. Cette situation expose les receveurs à des complications post-transfusionnelles, notamment infectieuses, qui pourraient être évitées. À cela s’ajoute une méfiance socioculturelle envers le don de sang, qui contribue à aggraver la pénurie de produits sanguins sûrs. Cette revue narrative a pour objectif de dresser un état des lieux des enjeux actuels liés à la sécurité transfusionnelle en ASS, avec le cas particulier de la RDC.

**Méthode:**

Une revue narrative intégrative a été menée entre janvier et avril 2025 à partir des bases de données (PubMed, Google Scholar), en utilisant des mots-clés en anglais et en français tels que: « transfusion safety », « sub-Saharan Africa », « blood transfusion risks », « post-transfusion surveillance », « transfusion-transmitted infections », « hémovigilance » et « Democratic Republic of the Congo », seuls ou combinés à l’aide d’opérateurs booléens. Des articles originaux, des revues systématiques, des rapports institutionnels et des recommandations d’organismes de santé publique, publiés entre 2000 et 2025, ont été inclus. La sélection a porté sur des sources pertinentes concernant les systèmes de sécurité transfusionnelle, les pratiques cliniques en contexte de ressources limitées et les modèles de surveillance en milieu à faible ou moyen de revenu.

**Résultats:**

La RDC ne dispose toujours pas d’un système national d’hémovigilance opérationnel. La gestion transfusionnelle reste décentralisée, fragmentée et insuffisamment standardisée, ce qui compromet la qualité et la sécurité des soins. L’absence de traçabilité et le dépistage parfois incomplet exposent les receveurs à des risques évitables. Toutefois, plusieurs initiatives locales émergent, soutenues par des organisations non gouvernementales (ONG), des institutions académiques ou des programmes internationaux. Parmi les leviers identifiés, figurent le développement d’outils numériques pour la surveillance, la mise en réseau de laboratoires sentinelles, l’amélioration de l’accès au sang sécurisé en périphérie, ainsi que la création de pôles régionaux de formation continue et de recherche appliquée en sécurité transfusionnelle.

**Conclusion:**

Pour garantir durablement la sécurité de la transfusion sanguine en RDC, une approche systémique, intégrée et multisectorielle est nécessaire, fondée sur l’innovation contextuelle, le soutien institutionnel et le renforcement des capacités locales de recherche. Pour mettre en place un système d’hémovigilance efficace, une mobilisation politique et un renforcement des partenariats sont indispensables.

## Abstract (en)

**Introduction:**

Ensuring the safety of blood transfusions remains a significant health challenge in sub-Saharan Africa (SSA), especially in the Democratic Republic of Congo (DRC). Political instability, inadequate infrastructure, and the absence of comprehensive hemovigilance systems exacerbate the risks. This situation exposes blood transfusion recipients to post-transfusion complications, especially infectious ones, that could be avoided. Additionally, sociocultural mistrust of blood donation contributes to the shortage of safe blood products. This narrative review aims to address the current issues related to transfusion safety in SSA, particularly in the DRC.

**Method:**

An integrative narrative review was conducted from January to April 2025 using the following English and French keywords: “transfusion safety,” “sub-Saharan Africa,” “blood transfusion risks,” “post-transfusion surveillance,” “transfusion-transmitted infections,” “hemovigilance,” and “Democratic Republic of the Congo,” either alone or in combination using Boolean operators. Original articles, systematic reviews, institutional reports, and recommendations from public health organizations published between 2000 and 2025 were included. The selection focused on sources relevant to transfusion safety systems, clinical practices in settings with limited resources, and surveillance models in low-and middle-income countries.

**Results:**

The DRC still lacks an operational national hemovigilance system. Transfusion management remains decentralized, fragmented, and insufficiently standardized, which compromises the quality and safety of care.

The lack of traceability and incomplete screening sometimes expose recipients to avoidable risks. However, several local initiatives supported by non-governmental organizations (NGOs), academic institutions, or international programs are emerging. Identified levers include developing digital surveillance tools, networking sentinel laboratories, improving access to safe blood in peripheral areas, and creating regional centers for continuing education and applied research in transfusion safety.

**Conclusion:**

Ensuring the long-term safety of blood transfusions in the DRC requires a systemic, integrated, multisectoral approach based on contextual innovation, institutional support, and strengthening local research capacities. Political mobilization and strengthening partnerships are essential to establishing an effective hemovigilance system.

## Introduction

Dans les pays à ressources limitées, la transfusion sanguine est considérée comme un vecteur majeur de transmission d’agents infectieux, en raison de systèmes de sécurité transfusionnelle encore fragiles. En Afrique subsaharienne (ASS), malgré des efforts constants pour améliorer les pratiques transfusionnelles, le risque d’infection après une transfusion reste élevé, soulevant ainsi des questions sur la sécurité des patients [26,44]. L’ampleur du défi est illustrée par le taux élevé de séropositivité parmi les donneurs et la persistance de pratiques de dépistage inadaptées ou incomplètes [39]. Aussi, l’instabilité géopolitique, marquée par des conflits armés, des déplacements de population massifs et une pression accrue sur le système de santé, dans certains pays à l’instar de la RDC, aggrave les risques transfusionnels [25,30]. La population, déjà vulnérable, est exposée à la malnutrition, aux traumatismes, aux infections graves et aux complications gynéco-obstétricales qui sont fréquemment à l’origine d’urgences transfusionnelles [44,45,46]. Or, l’augmentation des besoins dépasse largement les capacités de dépistage jugées insuffisantes.

Alors que des systèmes robustes de vigilance transfusionnelle sont mis en place dans les pays à revenu élevé, les pays de l’ASS quant à eux subissent de multiples défaillances structurelles. Le sang transfusé n’est pas tracé, les données consolidées sur les incidents post-transfusionnels sont rares, les tests de dépistage fiables sont couverts de manière limitée, et les politiques nationales de surveillance à long terme sont insuffisantes [8,11,19]. Ces lacunes exposent les patients à un risque élevé d’infections transmissibles par le sang. Ces infections peuvent être causées par le VIH, les hépatites B et C, la syphilis ou le paludisme. D’autres agents pathogènes émergents, comme le virus de l’herpès humain de type 8 (HHV-8), peuvent également être transmis, parmi beaucoup d’autres [10,17].

La difficulté réside en premier lieu dans le faible nombre de publications méthodiques, l’inexistence de systèmes de signalement performants et l’absence d’études post-don. Ce manque de données constitue un obstacle majeur à la conception et à la mise en œuvre de stratégies de prévention ciblées et efficaces, rendant ainsi la lutte contre les maladies virales, parasitaires et bactériennes plus ardue. Des initiatives régionales ont été destinées à remédier à cette situation (création de banques de sang centralisées ou de plateformes de déclaration des effets indésirables). Prises en amont, elles auraient permis d’évaluer adéquatement le terrain et d’assurer une couverture homogène. La RDC en est un exemple illustratif. En dépit d’une demande croissante en transfusions due à une forte charge de morbidité infectieuse et maternelle, les structures transfusionnelles y demeurent largement décentralisées, sous-équipées et faiblement encadrées. Ce contexte souligne l’urgence d’une réflexion approfondie sur les spécificités et les perspectives de la surveillance post-transfusionnelle dans le pays.

Dans cette revue narrative, nous proposons donc une analyse critique du risque infectieux transfusionnel en ASS, en mettant en lumière les principales failles du système de surveillance posttransfusionnelle et en explorant des perspectives d’amélioration adaptées au contexte de la RDC.

## Méthodologie

Cette synthèse narrative, basée sur une approche descriptive et intégrative, a pour objectif de résumer les connaissances actuelles sur les risques infectieux liés à la transfusion sanguine en ASS, en mettant l’accent sur les enjeux de la surveillance post-transfusionnelle. Ce format a été choisi pour sa capacité à intégrer des données hétérogènes, à la fois scientifiques et opérationnelles, et pour sa capacité à répondre aux réalités complexes des contextes instables, notamment en RDC.

La recherche documentaire a été conduite entre janvier et avril 2025 à partir des bases de données PubMed et Google Scholar, en combinant des mots-clés en anglais et en français à l’aide d’opérateurs booléens (AND, OR). Les termes les plus utilisés incluent: « *transfusion safety* », « *sub-Saharan Africa* », « *blood transfusion risks* », « post-transfusion surveillance », « *transfusiontransmitted infections* », « hémovigilance » et « Democratic Republic of the Congo » et leurs équivalents en français. Les termes exploratoires comme « anémie » ou « techniques de dépistage » ont été testés, mais se sont révélés peu contributifs.

Les documents publiés entre 2000 et 2025, disponibles en texte intégral en anglais ou en français, et traitant des risques transfusionnels infectieux, de la sécurité du sang ou des systèmes d’hémovigilance ont été sélectionnés. Bien que centrée sur l’ASS, la revue a inclus des travaux issus d’autres régions lorsque ceux-ci présentaient des innovations ou des modèles transposables à des environnements similaires.

**Figure 1:**
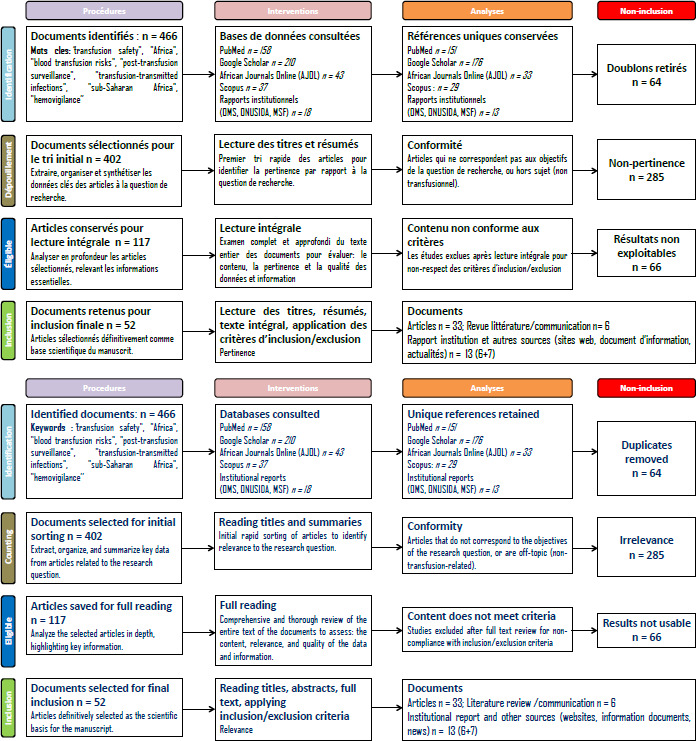
Processus de sélection des articles inclus dans la revue narrative

Outre les publications scientifiques (études originales, revues), des rapports institutionnels d’organisations reconnues (Organisation mondiale de la santé (OMS); Médecins sans frontières (MSF); Management Sciences for Health (MSH) et des sources en ligne spécialisées (Actualité. cd, Tout sur la transfusion) ont été examinés. Ces dernières ont été particulièrement utiles pour documenter les pratiques transfusionnelles dans des zones de conflit où les données scientifiques sont rares. Les publications à visée technique ou spécialisée, comme celles portant sur la génomique virale ou l’immunologie avancée, ont été exclues, sauf lorsqu’elles apportaient un éclairage direct sur les risques ou la surveillance post-transfusionnelle. La sélection des articles a suivi un processus rigoureux conforme aux recommandations PRISMA 2020, comme l’illustre la Figure 1. Une recherche systématique a été menée dans plusieurs bases de données électroniques, puis les doublons ont été éliminés après collecte. Les titres et résumés ont été examinés afin d’identifier les documents pertinents, selon des critères d’inclusion clairement définis. Les articles éligibles ont ensuite été soumis à une lecture intégrale afin d’évaluer leur validité scientifique et leur lien direct avec la problématique étudiée. Le processus global d’examen documentaire s’est articulé autour de deux axes complémentaires. Le premier, centré sur la sélection des études, a suivi les étapes classiques d’identification, de dépouillement, d’éligibilité et d’inclusion. Le second, basé sur l’analyse méthodologique, a permis d’interroger les procédures mobilisées, d’examiner les critères d’analyse retenus et de justifier les exclusions survenues après lecture intégrale. L’extraction finale des données s’est concentrée sur plusieurs éléments clés, notamment les pays concernés, les risques transfusionnels identifiés, les niveaux de mise en œuvre de la surveillance post-transfusionnelle et les recommandations formulées par les auteurs.

## Résultats

Le risque transfusionnel désigne l’ensemble des événements indésirables pouvant survenir chez un receveur de produits sanguins labiles, qu’ils soient d’origine infectieuse ou non. Ces risques varient en fonction du contexte épidémiologique, des pratiques transfusionnelles et des systèmes de sécurité en place. On distingue classiquement deux grandes catégories de risques: les risques infectieux et les risques non infectieux.

Les risques infectieux transfusionnels désignent la probabilité de transmission d’agents pathogènes par le sang ou ses composants lors d’une transfusion. Ils correspondent aux infections pouvant être contractées à travers ce geste médical, en lien avec la présence potentielle de virus, de bactéries, de parasites ou d’autres agents pathogènes dans le sang transfusé. En ASS, ces risques sont amplifiés par la forte prévalence de certaines infections dans la population générale [45]. Bien que théoriquement mieux encadrés, les donneurs bénévoles affiliés aux structures de santé demeurent exposés aux mêmes infections et deviennent de plus en plus rares. Cette rareté favorise le recours accru aux dons familiaux ou de remplacement, qui sont souvent associés à une sécurité moindre [18]. Ce contexte contribue à une qualité hétérogène du dépistage en laboratoire, ce qui compromet la sécurité des produits sanguins transfusés. Les agents infectieux les plus fréquemment rapportés sont classés selon leur évolution clinique en infections aiguës ou chroniques, chacune présentant des risques spécifiques:

- Les infections aiguës transmissibles par la transfusion: elles provoquent généralement des symptômes rapides après l’exposition et présentent un risque élevé dans les situations cliniques urgentes ou chez les patients vulnérables.
  - Paludisme *(Plasmodium spp)*: endémique en ASS, le paludisme constitue un risque transfusionnel majeur en raison de la fréquence élevée du portage asymptomatique du *Plasmodium* chez les donneurs de sang. Des études ont rapporté des taux de prévalence allant de 5 à plus de 50% selon les régions. Une enquête menée en RDC a révélé que les donneurs bénévoles à Kisangani, étaient pour 28,3% d’entre eux porteurs asymptomatiques du *Plasmodium* [7], tandis qu’en Ouganda, ce pourcentage a été estimé à environ 22% [41]. Ce risque est particulièrement élevé chez les receveurs non immunisés, notamment les jeunes enfants, les femmes enceintes et les patients immunodéprimés. En l’absence de dépistage systématique du *Plasmodium* dans les unités de sang, la transmission post-transfusionnelle du paludisme reste fréquente et souvent silencieuse, ce qui aggrave la morbidité dans ces groupes vulnérables [2,41].
  - Syphilis *(Treponema pallidum)*: cette infection bactérienne persiste dans les systèmes transfusionnels, en particulier lorsque les tests sérologiques sont inexistants ou mal interprétés. En l’absence de traitement approprié, elle peut évoluer rapidement et entraîner des complications graves [8].
  - Virus de l’herpès humain 8 (HHV-8): bien que rarement testé, ce virus, responsable du sarcome de Kaposi, est documenté comme potentiellement transmissible par transfusion dans certaines régions d’Afrique [20,12].

Bien que toutes ces infections puissent être transmises de façon aiguë par la transfusion, leur expression clinique diffère: le paludisme et la syphilis se manifestent souvent rapidement, tandis que le HHV-8 reste généralement latent, ce qui rend sa détection post-transfusionnelle plus difficile. L’ordre de présentation adopté ne reflète pas la fréquence exacte de ces infections dans les régions concernées, mais repose sur la disponibilité et la pertinence contextuelle des données pour la région d’Afrique centrale et de l’Est.

- Les infections chroniques transmissibles par la transfusion: ces agents infectieux s’installent dans le temps, entraînant des maladies à évolution lente, souvent graves, et difficilement réversibles.
  - Virus de l’immunodéficience humaine (VIH): malgré les avancées du dépistage, la transmission transfusionnelle demeure possible, en particulier lorsque les tests sérologiques utilisés en routine présentent une sensibilité inférieure à celle des techniques de biologie moléculaire telles que la réaction de polymérisation en chaîne (PCR). Ce déficit de performance, combiné à d’éventuelles erreurs humaines ou techniques au sein des centres de transfusion, contribue à maintenir un risque résiduel d’infections post-transfusionnelles [46].
  - Virus de l’hépatite B (VHB) et virus de l’hépatite C (VHC): très préoccupants en ASS en raison de leur forte prévalence et du manque d’outils de dépistage fiables ou disponibles, ces virus entraînent des hépatites chroniques avec risque de cirrhose ou de cancer hépatique [3].

Dans les pays où la sécurité transfusionnelle est élevée, les risques non infectieux sont désormais les plus fréquents. Cependant, en Afrique, ces risques sont souvent sous-estimés en raison d’une surveillance insuffisante, d’un manque de formation du personnel et de l’absence de systèmes de notification et d’analyse des effets indésirables. Parmi les plus fréquents, on distingue notamment:

- Les réactions immunologiques qui incluent les réactions hémolytiques (provoquées par des incompatibilités ABO ou Rh), les réactions non hémolytiques fébriles (liées à des anticorps anti-leucocytes présents chez le receveur) et les réactions allergiques (variant d’un simple prurit ou urticaire à des manifestations sévères comme l’anaphylaxie) [5,14,31].
- Chez les patients recevant des transfusions répétées sans traitement chélateur, l’accumulation de fer peut entraîner une hémochromatose. Cette complication progressive peut entraîner des lésions graves du foie, du cœur ou du système endocrinien [32].
- L’allo-immunisation érythrocytaire, qui correspond à la production d’anticorps dirigés contre des antigènes érythrocytaires non présents chez le receveur, est fréquente chez les polytransfusés, comme les patients drépanocytaires ou thalassémiques [34].

Ces risques soulignent l’importance de mettre en place un système d’hémovigilance structuré, capable de surveiller, signaler et prévenir efficacement les incidents transfusionnels à chaque étape de la chaîne, depuis le prélèvement jusqu’au suivi post-transfusionnel.

Bien que salvatrice dans de nombreuses situations cliniques critiques, la transfusion sanguine demeure un acte hautement risqué en ASS, en raison de la persistance d’un système de sécurité et de surveillance déficient. Cette section analyse trois points névralgiques: la couverture du dépistage, la traçabilité des produits sanguins et l’absence de systèmes structurés d’hémovigilance.

Dans plusieurs pays de l’ASS, la couverture des tests de dépistage reste insuffisante, tant en termes de disponibilité que de qualité, ce qui constitue un enjeu de santé publique majeur. Bien que la majorité des centres de transfusion revendiquent un dépistage systématique du VIH, du VHB, du VHC et de la syphilis, la réalité sur le terrain révèle d’importantes disparités régionales et structurelles.

Au Cameroun, en RDC et au Nigeria, des études ont révélé que certains centres de santé ont recours à des tests rapides de faible sensibilité, principalement dans les zones rurales et les régions en proie à des conflits [44].

Par ailleurs, la fenêtre sérologique demeure une limite majeure pour de nombreux agents infectieux, viraux, bactériens ou parasitaires, qui exposent les receveurs à des transmissions malgré des tests initiaux négatifs. Si de nos jours les techniques de biologie moléculaire permettent de raccourcir ce délai de détection, elles restent cependant marginales en pratique clinique. Ceci, en raison de leur coût et des contraintes techniques qui les limitent souvent aux protocoles de recherche [6,9, 22]. L’absence d’encadrement adéquat des donneurs bénévoles, pourtant considérés comme la source la plus sûre, les expose également à une forte prévalence d’infections. Ce manque de suivi favorise une certaine insouciance et entraîne une diminution progressive du volontariat. Dans les contextes d’urgence, cette pénurie conduit souvent à avoir recours à des dons non planifiés provenant de proches ou à des donneurs de remplacement, voire à rémunérer le donneur, ce qui fragilise davantage le système transfusionnel [4,43,50]. La traçabilité transfusionnelle, c’est-à-dire la capacité à relier un don de sang à un receveur et à retracer les incidents associés, est gravement compromise dans la majorité des systèmes transfusionnels des pays africains.

L’absence d’un système numérique centralisé entraîne des pertes d’information et rend quasi impossible toute enquête post-transfusionnelle efficace en cas d’effets indésirables [25].

En RDC, les dons de sang sont encore majoritairement gérés de manière décentralisée, sans registre national informatisé ni système d’identification sécurisé des unités transfusées. Cette absence de traçabilité rend difficile les rappels de lots contaminés et les enquêtes en cas de transmission confirmée, ce qui compromet ainsi la sécurité transfusionnelle [25].

L’hémovigilance, définie comme le dispositif de surveillance, d’analyse et de prévention des événements indésirables liés à la transfusion, est largement déficiente dans la majorité des pays d’ASS. Une enquête régionale de l’OMS indiquait que moins de 20% des pays disposaient d’un système fonctionnel, même si ce taux a atteint 49% en 2023. La majorité de ces dispositifs repose encore sur un signalement volontaire et non systématique, limitant leur capacité à prévenir efficacement les incidents transfusionnels [42]. Cette faiblesse serait aggravée par un manque de personnel formé, l’absence de cadre juridique contraignant et des procédures administratives lourdes, qui entravent le développement d’une véritable culture de la sécurité transfusionnelle. Même dans les pays disposant de plateformes de notification, comme le Rwanda ou le Sénégal, ces systèmes ne couvrent souvent qu’une partie du territoire, et les rapports sont rarement publiés ou intégrés à une stratégie nationale cohérente [28,49]. Par ailleurs, des cas d’infections posttransfusionnelles ont été signalés de manière sporadique. En Ouganda, une alerte concernant des cas de transmission du VHC a révélé des failles liées à un lot de tests de dépistage défectueux [33]. En RDC, des cas probables de transmission transfusionnelle du paludisme ont régulièrement été rapportés dans plusieurs hôpitaux, aussi bien en milieu urbain que rural, où le dépistage parasitaire est le plus souvent absent. Ce constat a motivé la réalisation d’une étude à Kisangani entre décembre 2012 et mars 2013, visant à estimer la prévalence du portage asymptomatique du *Plasmodium* chez les donneurs de sang bénévoles. L’enquête a révélé une prévalence de 28,3% parmi les donneurs du Centre provincial de transfusion sanguine, soulignant ainsi un risque important de transmission par voie transfusionnelle dans un contexte d’absence de dépistage systématique [7]. Toutefois, plusieurs études suggèrent que cette prévalence pourrait être sous-estimée en raison de l’utilisation d’outils diagnostiques à faible sensibilité. Au Mali, une enquête de 2019 a révélé un portage variant entre 6,5% et 74,1% selon les régions, tandis qu’au Niger, une étude menée en 2015 a rapporté une parasitémie à *Plasmodium* chez 67,5% des donneurs bénévoles de sang asymptomatique [23].

Ces observations soulignent l’urgence de mettre en place des systèmes d’hémovigilance plus rigoureux, d’assurer une surveillance épidémiologique continue, et de coordonner les efforts entre les laboratoires, les services cliniques, les autorités sanitaires et les centres de transfusion. Le renforcement de ces dispositifs, en incluant par exemple le dépistage systématique du paludisme et d’autres pathogènes ainsi que des outils numériques de traçabilité, apparaît indispensable pour prévenir efficacement les risques transfusionnels dans un contexte de vulnérabilité persistante. L’évaluation du risque transfusionnel ne saurait se limiter à une approche purement technique. Elle doit également prendre en compte les dynamiques épidémiologiques locales, en particulier la circulation d’agents infectieux émergents et l’augmentation de la vulnérabilité de certains groupes de patients. Le contexte épidémiologique en ASS est marqué par la coexistence de pathologies infectieuses endémiques, un système de soins sous-doté et une demande transfusionnelle élevée. Tout ceci confère à la transfusion une position ambivalente: salvatrice et potentiellement dangereuse.

Si les agents transfusionnels « classiques », comme le VIH, le VHB, le VHC ou la syphilis, restent prédominants, plusieurs études ont souligné la possible transmission par transfusion d’agents infectieux émergents ou négligés. Ces derniers sont souvent ignorés par les stratégies de la politique actuelle de dépistage.

Le HHV-8, responsable du sarcome de Kaposi, est fortement endémique dans certaines régions du monde. Des études ont montré une variation des taux de séroprévalence: 14% au Burkina Faso, 22% en République centrafricaine, 57% en Tanzanie, et entre 10 et 20% au Kenya et en Ouganda. Par ailleurs, une étude portant sur 991 receveurs de transfusion initialement séronégatifs a mis en évidence un excès de risque de séroconversion de 2,8% chez ceux ayant reçu du sang positif au HHV-8. (p < 0,05), principalement entre la 3^e^ et la 10^e^ semaine post-transfusionnelle [21,27,40].

Ces données soulignent l’importance d’un dépistage ciblé et d’une hémovigilance adaptée. En effet, la plupart des centres manquent d’outils diagnostiques et de systèmes de surveillance intégrés, ce qui compromet la prévention.

Les conséquences du risque transfusionnel sont particulièrement graves pour certains sousgroupes de patients, notamment les enfants, les femmes enceintes et les patients immunodéprimés (personnes vivant avec le VIH, patients cancéreux, transplantés). Chez ces individus une infection, même modérée, peut entraîner des complications graves, voire mortelles en raison des défenses immunitaires affaiblies.

- Chez l’enfant, la transfusion est souvent indiquée en contexte de paludisme grave, d’anémie hémolytique chronique, de syndrome hémorragique urémique ou de malnutrition sévère. Ces enfants, déjà immunodéprimés, présentent une susceptibilité accrue aux infections transmises, et les manifestations post-transfusionnelles peuvent être atypiques et évoluer rapidement vers des formes graves [35,47,51].
  - Chez les femmes enceintes lors de la délivrance en cas d’hémorragie obstétricale, la transfusion est souvent effectuée en urgence sans tests complets, dans des maternités non intégrées au circuit sécurisé du sang. Cette situation contribue à la transmission de pathogènes chroniques comme le VHB, avec des conséquences graves pour la mère et le fœtus [16,52].
  - Chez les personnes immunodéprimées, y compris les patients vivant avec le VIH ou sous chimiothérapie (qui reste rare en Afrique), le moindre agent infectieux transfusionnel peut déclencher des formes fulminantes de pathologies opportunistes. L’absence de suivi post-transfusionnel limite la détection précoce de ces événements, qui ont ainsi un impact sanitaire plus important [24,29].

Ces groupes à risque cumulent une exposition élevée et une capacité réduite à éliminer les agents infectieux transmis. Ces faits justifient la mise en place de protocoles transfusionnels spécifiques incluant un dépistage renforcé et un suivi systématique [13]. Le Tableau I, synthétise les groupes les plus exposés aux complications transfusionnelles en ASS, d’après les données disponibles.

**Tableau I:** Groupes à haut risque transfusionnel en Afrique subsaharienne Table I: High-risk groups for transfusion in sub-Saharan Africa

Groupe à risque	Facteurs de vulnérabilité	Conséquences potentielles
Femmes enceintes	Paludisme gestationnel, anémie sévère, risque de transmission verticale	Mort fœtale, prématurité, fausse couche, formes graves de paludisme
Enfants <5 ans	Anémie post-palustre, immunité immature	Réactions post-transfusionnelles sévères, infections chroniques
Immunodéprimés (VIH+)	Défense immunitaire altérée, fréquence élevée de transfusions	Réactivation virale, infections opportunistes, complications post-transfusionnelles
Victimes de guerre	Blessures hémorragiques urgentes, transfusions en contexte de crise	Sang non testé, transmission VIH/VHB/ VHC/syphilis/paludisme
Patients polytransfusés	Risque d’allo-immunisation, surcharge en fer, exposition cumulative aux pathogènes	Maladies chroniques post-transfusionnelles, surcharge hépatique

## Défis spécifiques à la République démocratique du Congo (RDC)

En RDC, la sécurité transfusionnelle est compromise par plusieurs facteurs, dont des problèmes structurels, contextuels et humains. Le système transfusionnel reste décentralisé et hétérogène, avec une faible supervision centrale, l’absence de normes harmonisées et une traçabilité encore largement déficiente. Peu de structures disposent de registres de suivi, de codification systématique des unités de sang ou de procédures d’alerte post-transfusionnelle. L’approvisionnement repose encore majoritairement sur des donneurs familiaux ou de remplacement, souvent issus de groupes à risque, ce qui accroît la probabilité de transmission d’agents infectieux tels que le VIH, le VHB, la syphilis ou le paludisme [18].

La spécificité du contexte congolais réside dans les conflits armés persistants, particulièrement dans les provinces de l’Est (Nord-Kivu, Sud-Kivu, Ituri), où les urgences massives (blessures de guerre, hémorragies obstétricales, violences sexuelles) génèrent une forte demande en sang, dans des conditions d’urgence souvent dépourvues de tests fiables [29,30,37]. Des ONG rapportent que dans les hôpitaux de Bukavu et Goma, des transfusions sont fréquemment effectuées sans dépistage complet, exposant les patients à un risque élevé de transmission [1,30].

Malgré quelques initiatives pilotes de l’OMS et de MSF visant à renforcer la traçabilité, cellesci sont restées limitées géographiquement et dans le temps. L’absence d’un système national d’hémovigilance, de formation continue et de mécanismes de supervision limite la capacité à prévenir et détecter les incidents post-transfusionnels. Le cas de la RDC illustre l’urgence d’intégrer durablement l’hémovigilance dans les politiques nationales, tout en tenant compte des réalités de terrain et du contexte humanitaire persistant. Face à la persistance des risques transfusionnels en ASS, plusieurs pistes émergentes pour structurer une médecine transfusionnelle plus intégrée, sécurisée et adaptée au contexte émergent sont envisageables. La mise en place de systèmes d’hémovigilance nationaux, inspirés de la pharmacovigilance [15,36,48], permettrait de détecter, signaler et analyser les événements post-transfusionnels. Des modèles pilotes intégrant des registres numériques, une traçabilité post-don et une coordination multisectorielle ont démontré leur efficacité, comme l’ont montré des études de cas au Sénégal et en Afrique du Sud [38]. Toutefois, leur interopérabilité reste limitée, notamment dans les zones rurales ou en situation d’urgence humanitaire.

La RDC a besoin de mesures spécifiques en raison de son contexte conflictuel et de ses faiblesses structurelles. Il est urgent d’y établir une autorité nationale autonome de régulation transfusionnelle, système d’hémovigilance fonctionnel et une plateforme numérique centralisée pour la déclaration des incidents [30,37]. Dans ce contexte instable, la collecte de sang sécurisé à partir de donneurs volontaires réguliers, le déploiement de tests rapides pour le VIH, le VHB, la syphilis ou le paludisme, ainsi que des kits transfusionnels mobiles [29], sont des priorités.

Enfin, l’intégration du risque transfusionnel dans les politiques nationales de santé publique, en particulier les programmes de santé maternelle, de lutte contre les infections transmissibles et les réponses d’urgence, s’inscrit pleinement dans l’approche proposée par l’OMS pour une santé transfusionnelle universelle [38]. Toutefois, l’instabilité sécuritaire persistante, notamment dans les zones de conflit actif, constitue un obstacle majeur à la mise en œuvre de ces stratégies. Dans ce contexte, l’adoption d’un plan stratégique quinquennal, assorti d’indicateurs clairs et de financements pérennes, constituerait une avancée significative, à condition que les actions prévues tiennent compte des contraintes liées à l’insécurité et à la désorganisation des systèmes de santé.

## Conclusion

La sécurité transfusionnelle en Afrique subsaharienne demeure un défi sanitaire majeur, résultant de facteurs structurels, techniques et contextuels. Les risques infectieux, toujours préoccupants, sont amplifiés par l’insuffisance de systèmes de dépistage fiables, l’absence de traçabilité du sang transfusé et la faiblesse des mécanismes d’hémovigilance. Cette vulnérabilité s’aggrave en situation de conflit armé ou de crise humanitaire, comme en RDC où les besoins transfusionnels explosent en raison des traumatismes balistiques et des hémorragies obstétricales, sans que les protocoles de sécurité ne soient systématiquement appliqués. Dans ce contexte, une approche systémique, intégrée et adaptée aux réalités locales est essentielle. Il faut dépasser les actions ponctuelles pour élaborer des politiques cohérentes, fondées sur des données locales, et soutenues par des outils de surveillance efficaces, ainsi que par une stratégie de santé publique globale.

La recherche scientifique, en particulier celle conduite par les acteurs locaux, peut jouer un rôle structurant. En documentant les réalités du terrain, en générant des données robustes et en proposant des solutions innovantes, elle deviendra un levier essentiel pour améliorer durablement la sécurité transfusionnelle et permettre l’élaboration d’approches adaptées, culturellement acceptables et logistiquement réalisables.

## Source de financement

Cette revue narrative n’a bénéficié d’aucun financement institutionnel ou privé. Elle a été menée de manière indépendante dans le cadre d’un travail académique, sans conflit d’intérêts financiers ni soutien externe. Les auteurs ont assumé les frais liés à l’accès aux ressources documentaires et à l’analyse.

## Contribution des auteurs et autrices

Lambert Morisho MULAKWA: conception de l’étude, rédaction

Archippe Muhandule BIRINDWA: révision Chérone Nancy Mbani MPEGA NTIGUI: correction du manuscrit et révisons Patrick Ntagereka BISIMWA (PNB): révision Sandrine Lydie OYEGUE LIABAGUI: correction du manuscrit, révision et supervision L’article a été lu et validé par tous les auteurs avant sa soumission.

## Conflit d’intérêt

Aucun conflit d’intérêts n’a été déclaré.

## References

[2] Ahmadpour E, Foroutan-Rad M, Majidiani H, Moghaddam SM, Hatam-Nahavandi K, Hosseini SA, Rahimi MT, Barac A, Rubino S, Zarean M, Mathioudakis AG, Cevik M (2019). Transfusion-Transmitted Malaria: A Systematic Review and Meta-analysis. Open Forum Infect Dis.

[3] Alassan KS, Imorou RS, Sonombiti H, Salifou K, Ouendo EM (2019). Séroprévalence et facteurs associés à l’hépatite virale B chez les gestantes à Parakou en République du Bénin. Pan Afr Med J.

[4] Allain JP (2011). Moving on from voluntary non-remunerated donors: who is the best blood donor?. Br J Haematol.

[5] Anani LY, Lafia E, Ahlonsou F, Sogbohossou P, Bigot A, Fagbohoun J, Meton A, Adjaka A, Latoundji S, Py JY, Zohoun IS (2014). Évaluation du groupage sanguin dans les systèmes ABO et Rh dans les formations sanitaires du Bénin. Transfus Clin Biol.

[6] Attou MA, Morand-Joubert L. Fiabilité du test rapide d’orientation diagnostique de l’infection à VIH: expérience au laboratoire de virologie de l’hôpital Saint-Antoine (Paris). Immuno-Anal Biol Spe.

[7] Bassandja JO, Agasa SB, Likwela JL (2014). Prévalence du portage asymptomatique du Plasmodium chez les donneurs bénévoles de sang à Kisangani, République démocratique du Congo. Pan Afr Med J.

[8] Bloch EM, Vermeulen M, Murphy E (2012). Blood transfusion safety in Africa: a literature review of infectious disease and organizational challenges. Transfus Med Rev.

[9] Borde C, Maréchal V, Barnay-Verdier S (2009). Apport de la biologie moléculaire dans l’identification de nouveaux virus. Rev Francoph Lab.

[10] Buerger CS, Jain H (2025). Infectious Complications of Blood Transfusion. 2023 Jul 31. In: StatPearls. Treasure Island (FL): StatPearls Publishing.

[11] Candotti D, Tagny-Tayou C, Laperche S (2021). Challenges in transfusion-transmitted infection screening in Sub-Saharan Africa. Transfus Clin Biol.

[12] Cappellini MD, Cohen A, Porter J, Taher A, Viprakasit V (2014). Guidelines for the Management of Transfusion Dependent Thalassaemia (TDT). 3rd edition. Nicosia (CY): Thalassaemia International Federation.

[14] Connan L (2019). La RFNH ou l’approche clinique d’une hyperthermie. Transfus Clin Biol.

[15] Dahourou H, Tapko JB, Nébié Y, Kiénou K, Sanou M, Diallo M, Barro L, Murphy E, Lefrère JJ (2012). Mise en place de l’hémovigilance en Afrique subsaharienne. Transfus Clin Biol.

[16] Deneux-Tharaux C, Bonnet MP, Tort J (2014). Épidémiologie de l’hémorragie du post-partum. J Gynecol Obstet Biol Reprod (Paris).

[17] Dosunmu AO, Akinbami AA, Ismail AK, Olaiya MA, Uche EI, Aile IK (2017). The cost-effectiveness of predonation screening for transfusion transmissible infections using rapid test kits in a hospital-based blood transfusion centre. Niger Postgrad Med J.

[18] Fonteyne G (2006). Enquête sur les perceptions du don bénévole de sang: Positionnement et enjeu d’une recherche. Civilisations. 1 avr.

[19] Garraud O, Filho LA, Laperche S, Tayou-Tagny C, Pozzetto B (2016). The infectious risks in blood transfusion as of today - A no black and white situation. Presse Med.

[20] Gobbini F, Owusu-Ofori S, Marcelin AG, Candotti D, Allain JP (2012). Human herpesvirus 8 transfusion transmission in Ghana, an endemic region of West Africa. Transfusion.

[21] Hladik W, Dollard SC, Mermin J, Fowlkes AL, Downing R, Amin MM, Banage F, Nzaro E, Kataaha P, Dondero TJ, Pellett PE, Lackritz EM (2006). Transmission of human herpesvirus 8 by blood transfusion. N Engl J Med.

[22] Houzé S, Paris L (2015). Apport des tests de diagnostic rapide en parasitologie: intérêt et limites. Rev Franc Lab.

[23] Iro A, Lamine MM, Lazoumar RH, Alkassoum I, Maman D, Laouali HAM, Doutchi M, Maiguizo S, Laminou IM (2019). Transfusional Malaria and Associated Factors at the National Blood Transfusion Center of Niamey-Niger. J Trop Med.

[24] Joly V (1997). Prophylaxie des infections chez les immunodéprimés: prévention des infections post-transfusionnelles. Ann Med Interne (Paris).

[25] Kabinda Maotela J, Ramazani SY, Misingi P, Dramaix-Wilmet M (2015). Transfusion sanguine en République démocratique du Congo: efforts réalisés et défis à relever. Med Sante Trop.

[26] Loua A, Nikiéma JB, Sougou A, Kasilo OMJ (2019). Transfusion en Afrique subsaharienne. Transfus Clin Biol.

[27] Malonga GA, Dienta S, Traore FT, Maiga Z, Ba A, Faye O, Chicaud E, Marot S, Calvez V, Marcelin AG, Jary A, Maiga AI (2022). Human Herpesvirus 8 seroprevalence among blood donors in Mali. J Med Virol.

[29] Marec-Berard P, Blay JY, Schell M, Buclon M, Demaret C, Ray-Coquard I (2003). Risk model predictive of severe anemia requiring RBC transfusion after chemotherapy in pediatric solid tumor patients. J Clin Oncol.

[31] Mertes PM, Boudjedir K (2013). Allergie et transfusion. Transfus Clin Biol.

[32] Murray C, De Gelder T, Pringle N, Johnson JC, Doherty M (2016). Gestion de la surcharge en fer auprès des patients en hématologie et en oncologie: répercussions sur la pratique. Can Oncol Nurs J.

[33] Nankya-Mutyoba J, Apica BS, Otekat G, Kyeyune DB, Nakyagaba L, Nabunje J, Nakafeero M, Seremba E, Ocama P (2021). Hepatitis C in Uganda: Identification of infected blood donors for micro-elimination. J Virus Erad.

[34] Natukunda B, Schonewille H, Ndugwa C, Brand A (2010). Red blood cell alloimmunization in sickle cell disease patients in Uganda. Transfusion.

[35] Nguefack F, Chelo D, Tejiokem MC, Pondy A, Njiki kinkela MJ, Dongmo R, Awa HD, Taguebue J, Guemkam G, Vougmo Meguejio Njua C, Ndombo PO (2012). Fréquence des anémies sévères chez les enfants âgés de 2 mois à 15 ans au Centre Mère et Enfant de la Fondation Chantal Biya, Yaoundé, Cameroun. Pan Afr Med J.

[36] Nsimba M (2018). Étude observationnelle sur l’hémovigilance transfusionnelle à Kinshasa, République Démocratique du Congo.

[40] Operskalski EA (2012). HHV-8, transfusion, and mortality. J Infect Dis.

[41] Owusu-Ofori A, Owusu-Ofori S, Bates I (2015). Transfusion-transmitted Malaria in Sub-Saharan Africa. ISBT Sci Ser. avr.

[42] Samukange WT, Kluempers V, Porwal M, Mudyiwenyama L, Mutoti K, Aineplan N, Gardarsdottir H, Mantel-Teeuwisse AK, Nuebling CM (2021). Implementation and performance of haemovigilance systems in 10 sub-saharan African countries is sub-optimal. BMC Health Serv Res.

[43] Schnuriger A, Dominguez S, Valantin MA, Tubiana R, Duvivier C, Ghosn J, Simon A, Katlama C, Thibault V (2006). Intérêt d’un nouveau test combiné antigène-anticorps pour le dépistage de l’infection par le virus de l’hépatite C: réduction de la fenêtre sérologique au cours de l’hépatite C aiguë chez le sujet co-infecté par le VIH. Pathol Biol (Paris).

[44] Tagny CT, Mbanya D, Tapko JB, Lefrère JJ (2008). Blood safety in Sub-Saharan Africa: a multi-factorial problem. Transfusion.

[45] Tagny CT, Murphy EL, Lefrère JJ (2014). Recherches Transfusionnelles en Afrique francophone. Le groupe de recherches transfusionnelles d’Afrique francophone: bilan des cinq premières années. Transfus Clin Biol.

[46] Tagny CT, Owusu-Ofori S, Mbanya D, Deneys V (2010). The blood donor in sub-Saharan Africa: a review. Transfus Med.

[47] Thiongane A, Ndongo AA, Ba ID, Boiro D, Faye PM, Keita Y, Ba A, Cissé DF, Basse I, Thiam L, Ly ID, Niang B, Ba A, Fall AL, Diouf S, Ndiaye O, Ba M, Sarr M (2016). Syndrome hémolytique et urémique de l’enfant au Centre hospitalier universitaire (CHU) de Dakar: à propos de quatre observations. Pan Afr Med J..

[50] Valerian DM, Mauka WI, Kajeguka DC, Mgabo M, Juma A, Baliyima L, Sigalla GN (2018). Prevalence and causes of blood donor deferrals among clients presenting for blood donation in northern Tanzania. PLoS One.

[51] Williams TN (2016). Sickle Cell Disease in Sub-Saharan Africa. Hematol Oncol Clin North Am.

[52] Wondmeneh TG, Mekonnen AT (2024). Epidemiology of hepatitis B virus infection among pregnant women in Africa: a systematic review and meta-analysis. BMC Infect Dis.

