# Supplementary material for: Transfusion risk in sub-Saharan Africa: current situation and challenges specific to the Democratic Republic of Congo
Source: Med Trop Sante Int. 2025 Sep 25;5(4):mtsi.v5i4.2025.693. [Article in French] doi: 10.48327/mtsi.v5i4.2025.693 (PMC12948373; doi:10.48327/mtsi.v5i4.2025.693)
Supplement: Related texts

# Research article: Transfusion risk in sub-Saharan Africa: current situation and challenges specific to the Democratic Republic of Congo

## Introduction

In countries with limited resources, blood transfusions are considered a major vector for transmitting infectious agents due to fragile transfusion safety systems. In sub-Saharan Africa (SSA), despite ongoing efforts to improve transfusion practices, the risk of infection remains high, raising questions about patient safety [26,44]. The scale of the challenge is illustrated by the high rate of HIV positivity among donors and the persistence of inadequate or incomplete screening practices [39]. Furthermore, geopolitical instability marked by armed conflict, mass population displacement, and increased pressure on the health system in certain countries, such as the DRC, exacerbates transfusion risks [25,30]. The already vulnerable population is exposed to malnutrition, trauma, serious infections, and gynecological and obstetric complications, which frequently cause transfusion emergencies [44,45,46]. However, the increase in demand far exceeds screening capacity, which is considered insufficient.

While high-income countries have robust transfusion safety systems in place, countries in SSA suffer from multiple structural failures. Transfused blood is not traced, data on post-transfusion incidents is scarce, reliable screening tests are limited, and national long-term surveillance policies are inadequate [8,11,19]. These shortcomings expose patients to a high risk of blood-borne infections. These infections can be caused by HIV, hepatitis B or C, syphilis, or malaria. Other emerging pathogens, such as human herpesvirus 8 (HHV-8), can also be transmitted [10,17].

The difficulty lies in the low number of systematic publications, ineffective reporting systems, and absence of post-donation studies. This lack of data poses a significant challenge to designing and implementing targeted, effective prevention strategies, hindering the fight against viral, parasitic, and bacterial diseases. Regional initiatives have been launched to address this issue, such as the creation of centralized blood banks and platforms for reporting adverse events. Had these initiatives been implemented earlier, they would have enabled us to adequately assess the situation and ensure uniform coverage.

The DRC is a case in point. Despite the growing demand for transfusions due to high levels of infectious and maternal morbidity, transfusion facilities there remain largely decentralized, under-equipped, and poorly supervised. This situation underscores the urgent need for indepth reflection on the specifics and prospects of post-transfusion surveillance in the country. In this narrative review, we propose a critical analysis of the risk of transfusion-related infection in SSA, highlighting the main flaws in the post-transfusion surveillance system and exploring prospects for improvement adapted to the DRC’s context.

## Methodology

This narrative synthesis is based on a descriptive and integrative approach. It aims to summarize the current knowledge on infectious risks associated with blood transfusions in SSA, emphasizing the challenges of post-transfusion surveillance. This format was chosen for its ability to integrate both scientific and operational heterogeneous data and respond to the complex realities of unstable contexts, particularly in the DRC.

A literature review was conducted from January to April 2025 using PubMed and Google Scholar, combining English and French keywords with Boolean operators (AND, OR). The most frequently used terms included: “transfusion safety,” “sub-Saharan Africa,” “blood transfusion risks,” “post-transfusion surveillance,” “transfusion-transmitted infections,” “hemovigilance,” and “Democratic Republic of the Congo,” as well as their French equivalents. Exploratory terms such as “anemia” and “screening techniques” were tested but proved to be of little use.

Documents published between 2000 and 2025 that were available in full text in English or French and dealing with infectious transfusion risks, blood safety, or hemovigilance systems were selected. Although the review focused on SSA, it included work from other regions when the work presented innovations or models that could be applied to similar environments.

In addition to scientific publications (original studies and reviews), we reviewed institutional reports from recognized organizations, such as the World Health Organization (WHO), Médecins Sans Frontières (MSF), and Management Sciences for Health (MSH), as well as specialized online sources, such as *Actualité.cd* and *Tout sur la transfusion.* The latter were particularly useful for documenting transfusion practices in conflict zones, where scientific data is scarce. Technical or specialized publications, such as those on viral genomics or advanced immunology, were excluded unless they provided direct insight into risks or post-transfusion monitoring.

The selection of articles followed a rigorous process in accordance with the PRISMA 2020 recommendations, as illustrated in Figure 1. A systematic search was conducted in several electronic databases, and duplicates were eliminated after collection. Titles and abstracts were reviewed to identify relevant documents according to clearly defined inclusion criteria. Eligible articles were then read in full to assess their scientific validity and direct relevance to the issue under study. The document review process was structured around two complementary axes. The first axis focused on study selection and followed the standard steps of identification, screening, eligibility, and inclusion. The second axis, based on methodological analysis, allowed us to examine the procedures used, review the selected analysis criteria, and justify any exclusions made after a thorough reading. The final data extraction focused on several key elements, including the countries involved, identified transfusion risks, levels of post-transfusion monitoring implementation, and recommendations made by the authors.

**Figure 1:** Process for selecting articles included in the narrative review

## Results

Transfusion risk refers to any adverse event that may occur in a recipient of labile blood products, whether infectious or non-infectious. The specific risks vary depending on the epidemiological context, transfusion practices, and safety systems in place. There are two main categories of transfusion risk: infectious and non-infectious.

Infectious transfusion risks refer to the probability of pathogens being transmitted through blood or blood components during a transfusion. These risks correspond to infections that can be contracted through the transfusion procedure due to the potential presence of viruses, bacteria, parasites, or other pathogens in the blood being transfused. In SSA, these risks are amplified by the high prevalence of certain infections in the general population [45]. Although volunteer donors affiliated with health facilities are theoretically better supervised, they remain exposed to the same infections and are becoming increasingly rare. This scarcity encourages the greater use of family or replacement donations, which are often associated with lower safety [18]. This context contributes to inconsistent laboratory screening quality, which compromises the safety of transfused blood products. The most frequently reported infectious agents are classified as acute or chronic infections according to their clinical course, each presenting specific risks.

- Acute transfusion-transmissible infections generally cause rapid symptoms after exposure and present a high risk in urgent clinical situations or in vulnerable patients.
  -Malaria *(Plasmodium* spp.): Malaria is endemic in SSA and is a major transfusion risk due to the high frequency of asymptomatic *Plasmodium* carriage among blood donors. Depending on the region, studies have reported prevalence rates ranging from 5% to over 50%. A survey in the DRC revealed that 28.3% of volunteer blood donors in Kisangani were asymptomatic carriers of *Plasmodium* [7]. In Uganda, this percentage was estimated at around 22% [41]. This risk is particularly high for non-immune recipients, especially young children, pregnant women, and immunocompromised patients. Without systematic screening of blood units for *Plasmodium,* post-transfusion malaria transmission remains common and often asymptomatic, exacerbating morbidity in these vulnerable groups [2, 41].
  -Syphilis *(Treponema pallidum):* This bacterial infection can persist in transfusion systems, especially when serological tests are unavailable or misinterpreted. Without appropriate treatment, it can progress rapidly and lead to serious complications [8].
  -Human herpesvirus 8 (HHV-8): Although it is rarely tested for, this virus that causes Kaposi’s sarcoma, has been documented as potentially transmissible by transfusion in certain regions of Africa [20,12].
  -Human herpesvirus 8 (HHV-8): Although it is rarely tested for, this virus that causes Kaposi’s sarcoma, has been documented as potentially transmissible by transfusion in certain regions of Africa [20,12].

All of these infections can be transmitted acutely through transfusion; however, their clinical expression differs. Malaria and syphilis often manifest rapidly, while HHV-8 generally remains latent, making more difficult its post-transfusion detection. The order of presentation does not reflect the exact frequency of these infections in the regions concerned but is based on the availability and contextual relevance of data for Central and East Africa.

- Chronic transfusion-transmissible infections establish themselves over time, leading to slow-progressing, often serious, and difficult-to-reverse diseases.
  -Human immunodeficiency virus (HIV): Despite advances in screening, transmission through transfusion remains possible, particularly when routine serological tests are less sensitive than molecular biology techniques, such as the polymerase chain reaction (PCR). This performance deficit, combined with potential human or technical errors in transfusion centers, contributes to maintaining a residual risk of post-transfusion infections [46].
  -Hepatitis B virus (HBV) and hepatitis C virus (HCV) are a major concern in SSA due to their high prevalence and the lack of reliable screening tools. These viruses cause chronic hepatitis, which carries a risk of cirrhosis or liver cancer [3].

In countries with high transfusion safety, non-infectious risks have become the most common. However, in Africa, these risks are often underestimated due to inadequate monitoring, insufficient staff training, and an absence of systems for reporting and analyzing adverse events. The most common risks include:

- Immunological reactions, including hemolytic reactions (caused by ABO or Rh incompatibilities), non-hemolytic febrile reactions (related to anti-leukocyte antibodies present in the recipient), and allergic reactions (ranging from pruritus or urticaria to anaphylaxis) [5,14,31].
- Without chelation therapy, repeated transfusions can lead to iron accumulation and hemochromatosis in patients. This progressive condition can cause serious damage to the liver, heart, and endocrine system [32].
- Erythrocyte alloimmunization, the production of antibodies against erythrocyte antigens absent in the recipient, is prevalent among patients receiving multiple transfusions, including those with sickle cell disease or thalassemia [34].

These risks underscore the need for a structured hemovigilance system that can effectively monitor, report, and prevent transfusion incidents at every stage, from collection to post-transfusion follow-up.

Although blood transfusions are life-saving in many critical clinical situations, they remain highly risky in SSA due to the persistence of deficient safety and surveillance systems. This section analyzes three key issues: screening coverage, traceability of blood products, and the lack of structured hemovigilance systems.

In several SSA countries, screening coverage is insufficient in terms of availability and quality, posing a significant public health concern. While most transfusion centers claim to screen for HIV, HBV, HCV, and syphilis systematically, significant regional and structural disparities exist in reality. In Cameroon, in DRC, and in Nigeria, studies have revealed that some health centers use rapid tests with low sensitivity, primarily in rural areas and regions affected by conflict [44]. Furthermore, the serological window period poses a significant limitation for many infectious agents, including viral, bacterial, and parasitic ones. This exposes blood transfusion recipients to potential transmission despite initial negative test results. Although molecular biology techniques can shorten this detection time, they are rarely used in clinical practice due to cost and technical constraints, which often limit their use to research protocols [6,9,22].

Despite being considered the safest source, volunteer donors are exposed to a high prevalence of infections due to the lack of adequate supervision. This lack of monitoring encourages carelessness and leads to a decline in volunteering. In emergency situations, this shortage often results in unplanned donations from relatives or replacement donors. It can even lead to the remuneration of donors, which further weakens the transfusion system [4, 43, 50].

Transfusion traceability—i.e., the ability to link a blood donation to a recipient and trace associated incidents—is severely compromised in most transfusion systems in African countries.

The absence of a centralized digital system results in lost information and renders in the event of adverse effects, effective post-transfusion investigations virtually impossible [25].

In the DRC, blood donations are mainly managed in a decentralized manner without a national computerized registry or secure identification system for transfused units. This lack of traceability makes it difficult to recall contaminated batches and investigate confirmed transmission cases, thereby compromising transfusion safety [25]. Hemovigilance, which is defined as the system for monitoring, analyzing, and preventing adverse events related to transfusion, is largely deficient in most SSA countries. A WHO regional survey indicated that fewer than 20% of countries had a functional system, though this figure increased to 49% in 2023. Most of these systems still rely on voluntary and nonsystematic reporting, which limits their ability to effectively prevent transfusion incidents [42]. This weakness is exacerbated by a lack of trained personnel, an absence of a binding legal framework, and cumbersome administrative procedures, all of which hinder the development of a genuine culture of transfusion safety.

Even in countries with reporting platforms, such as Rwanda and Senegal, these systems often cover only part of the territory. Reports are rarely published or integrated into a coherent national strategy [28, 49]. Additionally, cases of post-transfusion infections have been reported sporadically. In Uganda, an alert concerning HCV transmission revealed flaws related to a batch of defective screening tests [33].

In the DRC, probable cases of transfusion-transmitted malaria have been regularly reported in several hospitals, both urban and rural, where parasite screening is often absent. These reports prompted a study in Kisangani from December 2012 to March 2013, which aimed to estimate the prevalence of asymptomatic *Plasmodium* carriage among volunteer blood donors. The survey revealed a prevalence of 28.3% at the Provincial Blood Transfusion Center, indicating a significant risk of transfusion-transmitted malaria without systematic screening [7]. However, several studies suggest that this prevalence may be underestimated due to the use of diagnostic tools with low sensitivity. A 2019 survey in Mali revealed carriage rates ranging from 6.5% to 74.1%, depending on the region. In Niger, a 2015 study reported *Plasmodium* parasitemia in 67.5% of asymptomatic voluntary blood donors [23]. These observations underscore the urgent need to establish rigorous hemovigilance systems, ensure continuous epidemiological surveillance, and coordinate efforts between laboratories, clinical services, health authorities, and transfusion centers. Including systematic screening for malaria and other pathogens and digital traceability tools in these systems appears essential to effectively preventing transfusion risks in a context of persistent vulnerability.

The assessment of transfusion risk must extend beyond a purely technical approach. It must also consider local epidemiological dynamics, particularly the circulation of emerging infectious agents and the increased vulnerability of certain patient groups. SSA has an epidemiological context marked by the coexistence of endemic infectious diseases, an under-resourced healthcare system, and high demand for transfusions. This gives transfusion an ambivalent position: it can be life-saving yet also dangerous.

While “classic” transfusion-transmitted infections such as HIV, HBV, HCV, and syphilis remain predominant, several studies have highlighted the possible transmission of emerging or neglected infectious agents through transfusion. Current screening policy strategies often overlook these agents.

HHV-8, which causes Kaposi’s sarcoma, is highly endemic in certain regions of the world. Studies have shown varying seroprevalence rates: 14% in Burkina Faso, 22% in the Central African Republic, 57% in Tanzania, and 10-20% in Kenya and Uganda. Furthermore, a study of 991 initially seronegative transfusion recipients showed an excess seroconversion risk of 2.8% in those who received HHV-8-positive blood (p < 0.05), primarily between weeks three and ten post-transfusion [21,27,40].

These data underscore the importance of targeted screening and appropriate hemovigilance. However, most centers lack diagnostic tools and integrated surveillance systems, which compromises prevention efforts.

The consequences of transfusion risk are particularly serious for certain subgroups of patients, including children, pregnant women, and immunocompromised individuals, such as people living with HIV, cancer patients, and transplant recipients. For these individuals, even a moderate infection can lead to serious, potentially fatal, complications due to weakened immune defenses.

- In children, transfusions are often necessary for severe malaria, chronic hemolytic anemia, hemolytic uremic syndrome, and severe malnutrition. These children are immunocompromised and more susceptible to transmitted infections. Post-transfusion manifestations may progress rapidly to severe forms [35,47,51].
- During delivery, transfusions are often performed on an emergency basis without complete testing on pregnant women with obstetric hemorrhage in maternity wards that are not part of the secure blood supply system. This contributes to the transmission of chronic pathogens, such as HBV, with serious consequences for the mother and fetus [16,52].
- In immunocompromised individuals, including patients living with HIV or undergoing chemotherapy (which remains rare in Africa), even the slightest infectious agent transmitted through transfusion can trigger severe forms of opportunistic diseases. The lack of post-transfusion follow-up limits the early detection of these events, thus having a greater impact on health [24,29].

These at-risk groups have a high exposure level and a reduced ability to eliminate transmitted infectious agents. These facts justify implementing specific transfusion protocols, including enhanced screening and systematic follow-up [13]. Table I summarizes the groups most at risk of transfusion complications in SSA, based on available data.

**Table I:** High-risk groups for transfusion in sub-Saharan Africa

At-risk group	Vulnerability factors	Potential consequences
Pregnant women	Gestational malaria, severe anemia, risk of vertical transmission	Fetal death, prematurity, miscarriage, severe forms of malaria
Children under 5 years of age	Post-malarial anemia, immature immunity	Severe post-transfusion reactions, chronic infections
Immunocompromised individuals (HIV+)	Impaired immune defense, high frequency of transfusions	Viral reactivation, opportunistic infections, post-transfusion complications
War victims	Emergency hemorrhagic injuries, transfusions in crisis situations	Untested blood, transmission of HIV/HBV/HCV/syphilis/ malaria
Patients who have received multiple transfusions	Risk of alloimmunization, iron overload, cumulative exposure to pathogens	Chronic post-transfusion diseases, hepatic overload

## Specific challenges in the Democratic Republic of Congo (DRC)

In the DRC, several factors compromise transfusion safety, including structural, contextual, and human issues. The decentralized and heterogeneous transfusion system has weak central supervision, lacks harmonized standards, and has largely deficient traceability. Few facilities have tracking records, systematic blood unit coding, or post-transfusion alert procedures. The blood supply mainly relies on family or replacement donors, who are often from at-risk groups. This increases the likelihood of transmitting infectious agents, such as HIV, HBV, syphilis, and malaria [18].

The Congolese context is characterized by persistent armed conflicts, particularly in the eastern provinces (North Kivu, South Kivu, and Ituri), where mass emergencies (e.g., war wounds, obstetric hemorrhages, and sexual violence) generate a high demand for blood. Often, these emergencies occur in conditions where reliable testing is unavailable [29,30,37]. NGOs report that transfusions are frequently performed without comprehensive screening in hospitals in Bukavu and Goma, exposing patients to a high risk of transmission [1,30].

A few pilot initiatives aimed at strengthening traceability have been launched by the WHO and MSF, but they have remained limited in terms of geography and time. The absence of national hemovigilance systems, continuing education, and oversight mechanisms hinders the prevention and detection of post-transfusion incidents. The case of the DRC illustrates the urgent need to integrate hemovigilance into national policies in a sustainable way while considering the local situation and the ongoing humanitarian crisis. In the face of ongoing transfusion risks in SSA, there are several emerging approaches to establishing a more integrated, secure, and context-appropriate transfusion medicine system. Inspired by pharmacovigilance [15,36,48], the establishment of national hemovigilance systems would enable the detection, reporting, and analysis of post-transfusion events. Pilot models that incorporate digital registries, post-donation traceability, and multisectoral coordination have proven effective, as demonstrated by case studies in Senegal and South Africa [38]. However, their interoperability remains limited, particularly in rural areas or in humanitarian crisis.

The DRC requires tailored measures due to its conflict context and structural weaknesses. An autonomous national blood transfusion regulatory authority, a functional hemovigilance system, and a centralized digital platform for incident reporting are urgently needed [30,37]. In this unstable context, priorities include collecting safe blood from regular voluntary donors, deploying rapid tests for HIV, HBV, syphilis, and malaria, and providing mobile transfusion kits [29].

Finally, integrating transfusion risk into national public health policies, particularly those related to maternal health, communicable disease control, and emergency response, is fully in line with the WHO’s proposed approach to universal transfusion safety [38]. However, persistent security instability, particularly in areas of active conflict, poses a significant challenge to implementing these strategies. Adopting a five-year strategic plan with clear indicators and sustainable funding would be a significant step forward in this context, provided the planned actions account for the constraints associated with insecurity and disorganized health systems.

## Conclusion

Transfusion safety in sub-Saharan Africa is a significant health challenge resulting from structural, technical, and contextual factors. Infectious risks remain a concern and are amplified by the absence of reliable screening systems, the inability to trace transfused blood, and weak hemovigilance mechanisms. This vulnerability is further exacerbated in situations of armed conflict or humanitarian crisis. For example, in the DRC, transfusion needs are skyrocketing due to ballistic trauma and obstetric hemorrhage, yet safety protocols are not consistently implemented. In this context, an integrated, systemic approach adapted to local realities is essential. We must move beyond ad hoc actions and develop coherent policies based on local data, supported by effective surveillance tools, and backed by a comprehensive public health strategy.

Scientific research, particularly that conducted by local actors, can play a structuring role. By documenting local conditions, generating robust data, and proposing innovative solutions, scientific research will become an essential tool for improving transfusion safety sustainably and enabling the development of appropriate, culturally acceptable, and logistically feasible approaches.

## Funding

This narrative review did not receive any institutional or private funding. It was conducted independently as part of academic work without financial conflicts of interest or external support. The authors covered the costs of accessing documentary resources and conducting the analysis.

## Authors’ contributions

Lambert Morisho MULAKWA: study design, writing

Archippe Muhandule BIRINDWA: revision Cherone Nancy Mbani MPEGA NTIGUI: manuscript correction and revision Patrick Ntagereka BISIMWA: revision Sandrine Lydie OYEGUE LIABAGUI: Manuscript correction, revision, and supervision.

All authors read and approved the article prior to submission.

## Conflict of interest

The authors declare no conflicts of interest.
